# Association between polycystic ovary syndrome and non-infectious uveitis

**DOI:** 10.1038/s41598-022-27024-x

**Published:** 2023-01-06

**Authors:** Chae Eun Lee, Nang Kyung Lee, Christopher Seungkyu Lee, Suk Ho Byeon, Sung Soo Kim, Seung Won Lee, Yong Joon Kim

**Affiliations:** 1 Department of Ophthalmology, The Institute of Vision Research, Yonsei University College of Medicine, Yonsei-Ro 50-1, Seodaemun-Gu, Seoul, Republic of Korea; 2 Siloam Eye Hospital, Seoul, Republic of Korea; 3 Department of Precision Medicine, Sungkyunkwan University School of Medicine, Jangan-Gu Seobu-Ro 2066, Suwon, 16419 Korea

**Keywords:** Endocrinology, Medical research

## Abstract

Polycystic ovary syndrome (PCOS) is a common endocrine disease in young women. It has been reported that increased proinflammatory cytokines can induce systemic inflammation. However, the association between PCOS and uveitis remains elusive. In this study, we investigate the possible association between PCOS and uveitis using Korean National Health Insurance Service-National Sample Cohort. The incidence of non-infectious uveitis was compared between patients with and without PCOS before and after propensity score matching. Hazard ratios were determined using univariate and multivariate Cox regression models. Of 558,302 female participants, 2039 had PCOS and 8122 had non-infectious uveitis. The incidence of non-infectious uveitis was 35.1 per 10,000 person-years in the PCOS patients compared to 16.6 in non-patients (*P* < .001). This tendency remained after 1:3 propensity score matching. The hazard ratio of PCOS using a multivariate Cox regression model was 2.79 (95% CI, 1.92–4.05; *P* < .001) and 2.87 (95% CI, 1.77–4.67; *P* < .001) before and after matching, respectively. Our results suggests that PCOS is associated with non-infectious uveitis, particularly in women of reproductive age. This may be due to hormonal changes and proinflammatory factors. Future investigations should examine the clinical features and underlying mechanisms.

## Introduction

Uveitis is an inflammatory ocular disease involving any part of the uveal tract^1^. Its estimated incidence is 17–52 per 10,000 person-years and the estimated prevalence is 38–714 per 10,000 persons worldwide^2–4^. Uveitis can be categorized into infectious and non-infectious uveitis. Infectious uveitis can be due to tuberculosis, herpes virus, and toxoplasmosis. Most non-infectious uveitis is idiopathic, but some are related to autoimmune diseases such as Behçet’s disease, ankylosing spondylitis, and systemic lupus erythematosus^5^. With high proportion of patients having idiopathic uveitis, additional studies of systemic diseases causing non-infectious uveitis are needed.

Polycystic ovary syndrome (PCOS) is a common endocrine disease in young women. Chronic anovulation and hyperandrogenism are typical symptoms, and increased proinflammatory factors can induce systemic inflammation^6^. Emerging lines of evidence suggest that PCOS are associated with autoimmunity related to the hormonal changes^7^. Low progesterone levels in PCOS cause the immune system to be overstimulated, which increase the production of estrogen and creates a variety of autoantibodies. Anti-nuclear (ANA), anti-thyroid, anti-smooth muscle (SMA), anti-histone, anti-carbonic anhydrase, anti-ovarian, and anti-islet cell antibodies have been documented in PCOS. Systemic lupus erythematosus and Hashimoto thyroiditis has been reported as an autoimmune disease associated with PCOS^8^.

There are a few case reports of uveitis in patients with PCOS. One patient had three recurrent uveitis episodes within 1 year^9^. Another reported a woman with PCOS who was diagnosed with Vogt-Koyanagi-Harada disease, with a specific type of panuveitis^10^. Currently, the association between PCOS and uveitis remains unclear.

This study compared the incidence of non-infectious uveitis in women with and without PCOS using the South Korean National Health Insurance Service-National Sample Cohort (NHIS-NSC) 2002–2013. The prevalence rates of comorbidities including Behcet's disease, juvenile idiopathic arthritis, ankylosing spondylitis and systemic lupus erythematosus which are known to be related with uveitis were also investigated, particularly in those of reproductive age. This study may help to understand the pathogenesis of uveitis and its relationship with systemic diseases, including PCOS.

## Results

### Baseline characteristics of study population

This study evaluated 558,302 subjects, including 2039 patients diagnosed with PCOS. The mean age of the PCOS and non-PCOS groups was 28.6 ± 7.0 and 39.3 ± 22.7 years, respectively (*P* < 0.001). Since PCOS is a disease of women of reproductive age, most baseline characteristics and the prevalence of comorbidities differed significantly between the two groups. The incidence of non-infectious uveitis was 35.1 and 16.6 per 10,000 person-years in the PCOS and non-PCOS groups, respectively (*P* < 0.001). Table 1 summarizes the baseline characteristics of the study population.

**Table 1:** Baseline characteristics of women in cohort data.

	Entire cohort	PCOS	non PCOS	*P* value
Total no	558,302	2039	556,263	< .001
Non-infectious uveitis incidence per 10,000 person-years	16.6	35.1	16.6	
Age, mean (SD)	39.3 (22.7)	28.6 (7.0)	39.3 (22.7)	< .001
**Region of residence**				
Rural	261,709 (46.9)	1,044 (51.2)	260,665 (46.9)	.73
Urban	235,034 (42.1)	923 (45.3)	234,111 (42.1)	
Missing	61,559 (11.0)	72 (3.5)	61,487 (11.0)	
**Household income**				
0–30%	90,082 (16.1)	292 (14.3)	89,790 (16.1)	< .001
30–70%	163,619 (29.3)	745 (36.5)	162,874 (29.3)	
70–100%	243,042 (43.5)	930 (45.6)	242,112 (43.5)	
Missing	61,559 (11.0)	72 (3.5)	61,487 (11.1)	
Diabetes	61,861 (11.1)	158 (7.7)	61,703 (11.1)	< .001
Hypertension	117,953 (21.1)	96 (4.7)	117,857 (21.2)	< .001
Cardiovascular disease	58,471 (10.5)	53 (2.6)	58,418 (10.5)	< .001
Chronic kidney disease	29,023 (5.2)	79 (3.9)	28,944 (5.2)	.007
**Charlson Comorbidity index**				
0	102,915 (18.4)	302 (14.8)	102,613 (18.4)	< .001
1	158,563 (28.4)	553 (27.1)	158,010 (28.4)	
2 and more	296,824 (53.2)	1,184 (58.1)	295,640 (53.1)	
**Comorbidity**				
Behcet’s disease	860 (0.15)	7 (0.34)	853 (0.15)	.03
Juvenile idiopathic arthritis	217 (0.04)	5 (0.25)	212 (0.04)	< .001
Ankylosing spondylitis	3342 (0.60)	3 (0.15)	3339 (0.60)	.008
Systemic lupus erythematosus	1241 (0.22)	7 (0.34)	1234 (0.22)	.25
Ulcerative colitis	1691 (0.30)	7 (0.34)	1684 (0.30)	.74
Tuberculosis	8814 (1.58)	30 (1.47)	8784 (1.58)	.70
Sarcoidosis	75 (0.01)	0 (0)	75 (0.01)	.60
Crohn’s disease	1009 (0.18)	2 (0.10)	1,007 (0.18)	.38
Multiple sclerosis	178 (0.03)	2 (0.10)	176 (0.03)	.09
Reactive arthritis	1306 (0.23)	4 (0.20)	1302 (0.23)	.72

*PCOS* Polycystic ovary syndrome.

Data are presented as No. (%).

### Factors associated with non-infectious uveitis

Older age, rural residence, higher income, and comorbidities were associated with an increased risk of uveitis. In the univariate regression analysis, the hazard ratio (HR) of PCOS was 1.77 (95% CI, 1.22–2.57; *P* = 0.003), which is relatively low compared to other known comorbidities associated with uveitis. However, in the multivariate regression analysis adjusting for age, the HR of PCOS increased to 2.79 (95% CI, 1.92–4.05; *P* < 0.001), the fourth highest HR after those of sarcoidosis, Behçet’s disease, and JIA (Table 2).

**Table 2:** Univariate and multivariate cox regression model analysis of factors associated with non-infectious uveitis.

	Univariate		Multivariate	
	HR (95% CI)	*P* value	HR (95% CI)	*P* value
**Age group**				
10–19	1 (Reference)		1 (Reference)	
20–29	1.88 (1.64–2.15)	< .001	1.88 (1.64–2.16)	< .001
30–39	2.17 (1.90–2.47)	< .001	2.06 (1.81–2.36)	< .001
40–49	2.45 (2.16–2.78)	< .001	2.16 (1.90–2.45)	< .001
50 and more	6.64 (5.93–7.45)	< .001	4.27 (3.79–4.82)	< .001
**Region of residence**				
Rural	1.09 (1.05–1.14)	< .001	1.10 (1.05–1.15)	< .001
Urban	1 (Reference)		1 (Reference)	
**Household income**				
0–30%	0.95 (0.89–1.01)	.07	0.83 (0.78–0.88)	< .001
30–70%	0.87 (0.83–0.92)	< .001	0.90 (0.86–0.95)	< .001
70–100%	1 (Reference)		1 (Reference)	
**Comorbidity**				
Hypertension	3.72 (3.56–3.88)	< .001	1.59 (1.50–1.69)	< .001
Diabetes	3.38 (3.22–3.54)	< .001	1.43 (1.35–1.51)	< .001
Chronic kidney disease	2.61 (2.44–2.79)	< .001	1.26 (1.18–1.36)	< .001
PCOS	1.78 (1.22–2.57)	.003	2.79 (1.92–4.05)	< .001
Behcet’s disease	7.83 (6.40–9.60)	< .001	5.47 (4.43–6.76)	< .001
Juvenile idiopathic arthritis	2.56 (1.28–5.12)	.008	3.31 (1.58–6.94)	.002
Ankylosing spondylitis	3.51 (3.00–4.10)	< .001	1.62 (1.37–1.90)	< .001
Systemic lupus erythematosus	2.46 (1.83–3.30)	< .001	1.53 (1.13–2.06)	.005
Ulcerative colitis	1.76 (1.30–2.38)	< .001	1.03 (0.76–1.40)	.85
Tuberculosis	2.04 (1.80–2.31)	< .001	1.29 (1.13–1.46)	< .001
Sarcoidosis	11.37 (6.46–20.00)	< .001	6.06 (3.35–10.95)	< .001
Crohn’s disease	2.24 (1.58–3.16)	< .001	1.24 (0.86–1.79)	.24
Multiple sclerosis	2.75 (1.31–5.78)	.007	1.30 (0.58–2.89)	.52
Reactive arthritis	2.48 (1.86–3.31)	< .001	1.50 (1.13–2.00)	.006

*Ref* Reference, *PCOS* Polycystic ovary syndrome, *HR* Hazard ratio.

### Comorbidities in reproductive aged women with or without uveitis

Since PCOS is a disease of women of reproductive age, we compared the prevalence of comorbidities in subjects with or without uveitis in women aged 15–49 years. The prevalence of PCOS in the uveitis patient group was 1.05%, the fourth highest after Behçet’s disease, tuberculosis, and ankylosing spondylitis, which was significantly higher than in the non-uveitis patient group (*P* = 0.03). Table 3 gives the prevalence of other comorbidities.

**Table 3:** Prevalence of comorbidities in women of reproductive age with or without uveitis.

Comorbidity	No. (%)		*P* value
	Uveitis patients (n = 2634)	Non-uveitis patients (n = 279,048)	
PCOS	28 (1.05)	1,979 (0.71)	.03
Behcet’s disease	60 (2.28)	445 (0.16)	< .001
Juvenile idiopathic arthritis	1 (0.04)	124 (0.04)	.88
Ankylosing spondylitis	31 (1.18)	504 (0.18)	< .001
Systemic lupus erythematosus	22 (0.84)	672 (0.24)	< .001
Ulcerative colitis	10 (0.38)	672 (0.24)	.15
Tuberculosis	49 (1.86)	3,541 (1.27)	.007
Sarcoidosis	6 (0.23)	25 (0.01)	< .001
Crohn’s disease	4 (0.15)	355 (0.13)	.72
Multiple sclerosis	2 (0.08)	61 (0.02)	.06
Reactive arthritis	10 (0.38)	452 (0.16)	.006

Data are presented as No. (%).

### Data analysis after propensity score matching

Because the baseline characteristics of the PCOS and non-PCOS groups differed significantly, we analyzed the HR for the risk of uveitis after 1:3 propensity score matching of variables associated with uveitis. After propensity score matching, the PCOS and non-PCOS groups included 1966 and 5881 subjects, respectively. No major imbalances in the propensity-matched cohorts were observed when evaluated using standardized mean differences within groups (Table 4).

**Table 4:** Baseline characteristics of propensity score-matched cohorts.

	PCOS	non PCOS	SMD
Total no	1966	5881	0.01
Age, mean (SD)	28.51 (7.11)	28.65 (7.34)	− 0.008
**Region of residence**			
Rural	1044 (53.1)	3125 (53.1)	< − 0.001
Urban	922 (46.9)	2756 (46.9)	
Diabetes	153 (7.8)	426 (7.2)	− 0.02
Hypertension	92 (4.7)	256 (4.4)	− 0.01
Chronic kidney diseases	78 (4.0)	226 (3.8)	− 0.006
**Household income**			
0–30%	291 (14.8)	870 (14.8)	0.001
30–70%	745 (37.9)	2236 (38.0)	
70–100%	930 (47.3)	2775 (47.2)	

*PCOS* Polycystic ovary syndrome, *SMD* Standardized mean difference.

Data are presented as No. (%).

After matching, the overall incidence of non-infectious uveitis per 10,000 person-years was 36.7 and 9.1 in the PCOS and non-PCOS groups, respectively. The respective incidences of anterior uveitis were 2.88 and 0.83 and those of non-anterior uveitis were 0.79 and 0.13 in the PCOS and non-PCOS groups. In the matched cohort, the fully adjusted HR for overall uveitis was 2.87 (95% CI, 1.77–4.67; *P* < 0.001). The fully adjusted HR of non-anterior uveitis was 4.76 (95% CI, 1.50–15.09; *P* = 0.008), suggesting that PCOS increased the risk of non-anterior uveitis compared to anterior uveitis. The tendency of the results was consistent in the minimally adjusted and additionally adjusted Cox multivariate regression models (Table 5).

**Table 5:** The hazard ratio of uveitis in PCOS patients after propensity score matching.

	HR (95% CI)					
	Minimally adjusted^a^	*P* value	Additionally adjusted	*P* value	Fully adjusted	*P* value
Non-infectious uveitis (n = 28)	2.87 (1.77–4.67)	< .001	2.89 (1.78–4.69)	< .001	2.87 (1.77–4.67)	< .001
Non-infectious anterior uveitis (n = 22)	2.43 (1.43–4.14)	.001	2.45 (1.44–4.17)	< .001	2.43 (1.43–4.14)	.001
Non-infectious non-anterior uveitis (n = 6)	4.72 (1.49–14.95)	.008	4.69 (1.48–14.86)	.009	4.76 (1.50–15.09)	.008

^a^Minimally adjusted: age; Additionally adjusted: age, region of residence, household income; Fully adjusted: age, region of residence, household income, comorbidities (autoimmune)^b^.

^b^Comorbidities: Behcet’s disease, Juvenile idiopathic arthritis, Ankylosing spondylitis, Systemic lupus erythematosus, Ulcerative colitis, Tuberculosis, Sarcoidosis, Crohn’ disease, Multiple sclerosis, Reactive arthritis.

## Discussion

Although several autoimmune diseases are closely related to non-infectious uveitis, studies have not fully investigated PCOS and uveitis. This study demonstrates an association between PCOS and uveitis based on a long-term national cohort study in South Korea.

In the multivariate Cox regression analysis adjusted for age, the HR of all comorbidities except PCOS and JIA decreased. This correlates with the fact that the two diseases mainly occur at a young age, while the incidence of uveitis usually increases with age^4^. In a similar context, we evaluated the prevalence of comorbidities with women of reproductive age (15–49 years), when PCOS mainly occurs. The prevalence of PCOS was 1.05%, the fourth highest after Behçet’s disease, tuberculosis, and ankylosing spondylitis. Therefore, the effect of PCOS on uveitis may have been underestimated in the general population.

PCOS often accompanies systemic inflammation. In a previous meta-analysis of inflammatory markers in PCOS patients, circulating C-reactive protein (CRP) was significantly elevated independent of obesity, leading to a chronic inflammatory condition^11^. High anti-ovarian antibody concentrations were also found in PCOS patients, which might be related to abnormal cytokine production^12^. Other organ-specific antibodies and non-specific antibodies such as ANA and SMA were significantly high in PCOS patients, presenting possible autoimmunity of the disease^7^. In a case report on a PCOS patient with three recurrent uveitis episodes, the cortisol level, erythrocyte sedimentation rate, and CRP level were elevated, indicating systemic inflammation^9^ Therefore, we believe these inflammatory conditions in PCOS patients can induce non-infectious uveitis. Clinically, metformin is often used as an anti-inflammatory drug in PCOS patients^13,14^. Although insulin resistance was not evident in the patient in the above case report, she was given metformin as an anti-inflammatory agent^9^.

Female sex hormones seem to play major roles in the two diseases. PCOS is characterized by acyclic estrogen overexpression and low progesterone levels^6^. In a study of acute anterior uveitis (AAU) and the menstrual cycle, AAU attacks peaked in the postovulatory phase^15^. Since both hormones have anti-inflammatory effects, including in ocular diseases, the authors suggested that withdrawing either or both hormones provoked the onset of uveitis^16,17^. Similarly, the postpartum decrease in female hormones is thought to increase the risk of uveitis^18^. There was a case report on a PCOS patient with bilateral anterior uveitis after using clomiphene citrate, an anti-estrogen used to induce ovulation^19^. This also suggests the anti-inflammatory potential of female hormones in the development of uveitis.

Since this study is based on cohort data, there are limitations in retrospective study design and diagnosis errors. Therefore, only patients with two or more prescriptions for the same ICD-10 code were included and those diagnosed during the first two years were excluded to rule out recurrence. Also, there is a limitation to figure out exact severity of diseases in this study, but we minimized the confounders by propensity score matching of 7847 patients.

In conclusion, this is the first population-based study to investigate the relationship between PCOS and uveitis. PCOS is closely related to non-infectious uveitis in women of reproductive age. The balance between female sex hormones and proinflammatory factors seems to influence the occurrence of non-infectious uveitis in PCOS patients, but additional studies are needed to explain the pathogenesis of the two diseases.

## Methods

### Data source and study approval

This was a nationwide, retrospective, cohort study based on National Health Insurance Sharing Service-National Sample Cohort (NHISS-NSC), which comprises nationwide health insurance claims data covering approximately 2.2% of the population of Korea, and includes the beneficiaries’ general characteristics, all diagnoses, procedures, and treatments received in health care services, and inpatient and outpatient prescriptions. NHIS-NSC was built based on the data obtained from the Korean Health insurance claim data covering approximately 97% of total population of Korea and by stratifying age, gender, economic power and residential areas to represent the entire population^20^.

This study was approved by the Institutional Review Board (IRB)/Ethics Committee of Severance Hospital, Yonsei University Health System (IRB no. 4-2022-0031), which also waived the requirement for informed consent due to the retrospective study design and use of de-identified data. The research adhered to the tenets of the Declaration of Helsinki. All methods were performed in accordance with the relevant guidelines and regulations.

### Definition of disease

The primary study outcome was the incidence of non-infectious uveitis, which was subdivided into anterior and non-anterior uveitis. The ICD-10 codes for anterior and non-anterior non-infectious uveitis were from a previous study reporting the incidence and prevalence of uveitis in Korea^4^. A patient was defined operationally as having a disease when given the ICD-10 code for that disease at least twice, (Supplementary Table 1). The ICD-10 codes considered were those for PCOS (E28.2), Behçet’s disease (M35.2X), juvenile idiopathic arthritis (JIA; M08.X), ankylosing spondylitis (M45.X), systemic lupus erythematosus (M32.X), ulcerative colitis (K51.X), tuberculosis (A15.X, A16.X, A17.X, A18.X, or A19.X), sarcoidosis (D86.X), Crohn’s disease (K50.X), multiple sclerosis (G35.X), and reactive arthritis (M02.X). Information on diabetes, hypertension, chronic kidney disease, and the Charlson comorbidity index was obtained from a combined operational definition and general health examination, including self-reported questionnaires and personal medical interviews (Supplementary Table 2).

### Study population, sociodemographic factors and comorbidities

We identified 560,645 women in the NHIS-NSC. We excluded those with a history of PCOS or non-infectious uveitis during 2002 and 2003, leaving a final sample of 558,302 participants. Demographics such as age, region of residence, and household income were obtained from insurance eligibility data.

### Incidence of uveitis

To exclude recurrent cases, data from the first 2 years (2002–2003) were ignored. Time of the first incident was defined as the date of the first visit to an ophthalmologist for uveitis. For subjects with PCOS, follow-up started on the first day of PCOS diagnosis. For those without PCOS, follow-up started on 1 January 2004 and ended on the last day of NHIS coverage. For subjects with uveitis, the incident case person-years were counted until the time of the first incident.

### Propensity score matching

In the full unmatched cohort of 558,302 women, 1:3 matching between those with and without PCOS was performed to generate a 1:3 matched cohort of 7847 patients (PCOS 1966, non-PCOS 5881) matched by age, region of residence, diabetes, hypertension, chronic kidney disease, and household income, which have been reported to be associated with uveitis^4^.

### Statistical analysis

The chi-square test was used to compare the patients’ baseline characteristics in the full unmatched cohort (Tables 1 and 3). Univariate and multivariate Cox regressions were performed on the risk of uveitis for age group, region of resistance, household income, and comorbidities in the full unmatched cohort (Table 2)^21^. In the full unmatched cohort (n = 558,302), the index date for Cox regression was defined as the date of the initial uveitis diagnosis from the date of the initial PCOS diagnosis and initial inclusion of the cohort in cases of non-PCOS.

During 1:3 propensity score matching, the goodness of the match was confirmed by comparing the exposure-based propensity score density with the standardized difference (Table 4)^22^. In the 1:3 propensity score-matched cohort (n = 7847), the index date was defined as the date of the initial diagnosis of uveitis from the date of the initial diagnosis of PCOS and matched opponent inclusion date in cases of non-PCOS patients to reduce immortal time bias.

Incidence rates were calculated for the propensity score-matched cohort for the entire study period. In addition, a Cox regression was performed on total non-infectious, anterior, and non-anterior uveitis in the propensity score-matched cohort. The Cox regression analysis included minimally adjusted (age), additionally adjusted (age, region of residence, and household income), and fully adjusted (age, region of residence, household income, and comorbidities, including autoimmune diseases) models (Table 5).

The adequacy of each Cox regression was confirmed using log–log survival plots and Schoenfeld residuals. The statistical analyses were performed with R ver. 4.2.2 (R Foundation, Vienna, Austria) and SAS ver. 9.4 (SAS Institute, Cary, NC). A *P* value < 0.05 was considered statistically significant.

## Supplementary Information

Supplementary Information.

## Data Availability

The data that support the findings of this study are available from NHISS but restrictions apply to the availability of these data, which were used under license for the current study, and so are not publicly available. Data are however available from the authors upon reasonable request and with permission of NHISS.
