# Supplementary material for: Association between polycystic ovary syndrome and non-infectious uveitis
Source: Sci Rep. 2023 Jan 6;13:277. doi: 10.1038/s41598-022-27024-x (PMC9822991; doi:10.1038/s41598-022-27024-x)
Supplement: Supplementary file 1 — Supplementary Information. [file 41598_2022_27024_MOESM1_ESM.docx]

**Supplementary Materials for:**

Association between Polycystic Ovary Syndrome and Non-Infectious Uveitis

Chae Eun Lee, MD^1,2,*^; Nang Kyung Lee^3,*^; Christopher Seungkyu Lee, MD, PhD^1^; Suk Ho Byeon, MD, PhD^1^; Sung Soo Kim, MD^1^; Seung Won Lee, MD, PhD^3†^; Yong Joon Kim, MD, PhD^1,†^

^1^Department of Ophthalmology, Institute of Vision Research, Yonsei University College of Medicine, Seoul, Republic of Korea

^2^Siloam Eye Hospital, Seoul, Republic of Korea

^3^Department of Precision Medicine, Sungkyunkwan University School of Medicine, Suwon, Republic of Korea

^*^Chae Eun Lee and Nang Kyung Lee contributed equally to this work.

^†^Corresponding authors

**Supplementary Materials**

**Supplementary Table 1**. International Classification of Diseases 10^th^ Revision (ICD-10) Codes for Noninfectious Uveitis

**Supplemental Table 2**. International Classification of Diseases10^th^ Revision (ICD-10) Codes for Systemic Diseases

**Supplementary Table 1**. International Classification of Diseases 10^th^ Revision (ICD-10) Codes for Noninfectious Uveitis

| **Anterior uveitis** | |
| --- | --- |
| H20.0 | Acute and subacute iridocyclitis |
| H20.1 | Chronic iridocyclitis |
| H20.2 | Lens-induced iridocyclitis |
| H20.8 | Other iridocyclitis |
| H20.9 | Iridocyclitis, unspecified |
| H22.1 | Iridocyclitis in other diseases classified elsewhere |
|  | Iridocyclitis in ankylosing spondylitis |
|  | Iridocyclitis in sarcoidosis |
| H22.8 | Other disorders of iris and ciliary body in diseases classified elsewhere |
| **Non-anterior uveitis, including intermediate, posterior, or panuveitis** | |
| H30.0 | Focal chorioretinal inflammation |
|  | Focal chorioretinitis |
|  | Focal choroiditis |
|  | Focal retinitis |
|  | Focal retinochoroiditis |
| H30.1 | Disseminated chorioretinal inflammation |
|  | Disseminated chorioretinitis |
|  | Disseminated choroiditis |
|  | Disseminated retinitis |
|  | Disseminated retinochoroiditis |
| H30.2 | Posterior cyclitis |
|  | Pars planitis |
| H30.8 | Other chorioretinal inflammations |
|  | Harada’s diseases |
| H30.9 | Chorioretinal inflammation |
|  | Chorioretinitis NOS |
|  | Choroiditis NOS |
|  | Retinitis NOS |
|  | Retinochoroiditis NOS |
| H35.05 | Retinal vasculitis |
|  | Retinal perivasculitis |

**Supplementary Table 2**. International Classification of Diseases10^th^ Revision (ICD-10) Codes for Systemic Diseases

| **Disease** | **ICD-10** |
| --- | --- |
| Diabetes | E10.1, E10.5, E10.9, E11.1, E11.5, E11.9, E13.1, E13.5, E13.9, E14.1, E14.5, E149 |
| Hypertension | I10.X, I11.X, I12.X |
| Cardiovascular disease | I00.X, I01.X, I02.X, I05.X, I06.X, I07.X, I08.X, I09.X, I20.X, I21.X, I22.X, I23.X, I24.X, I25.X, I26.X, I27.X, I28.X, I30.X, I31.X,I32.X, I33.X, I34.X, I35.X, I36.X, I37.X, I38.X, I39.X, I40.X, I41.X, I42.X, I42.X, I43.X, I44.X, I45.X, I50.X, I51.X, I52.X, I239, I529 |
| Chronic kidney disease | E102, E112, E132, E142, , I120, M200, M313, M319, M321B, N02.X, N03.X, N04.X, N05.X, N06.X, N07.X, N08.X, N11.X, N12.X, N14.X, N18.X, N19.X, N26.X, N158, N159, N160, N162, N163, N164, N168, Q61.2, Q61.3, Q61.5, Q61.9 |
